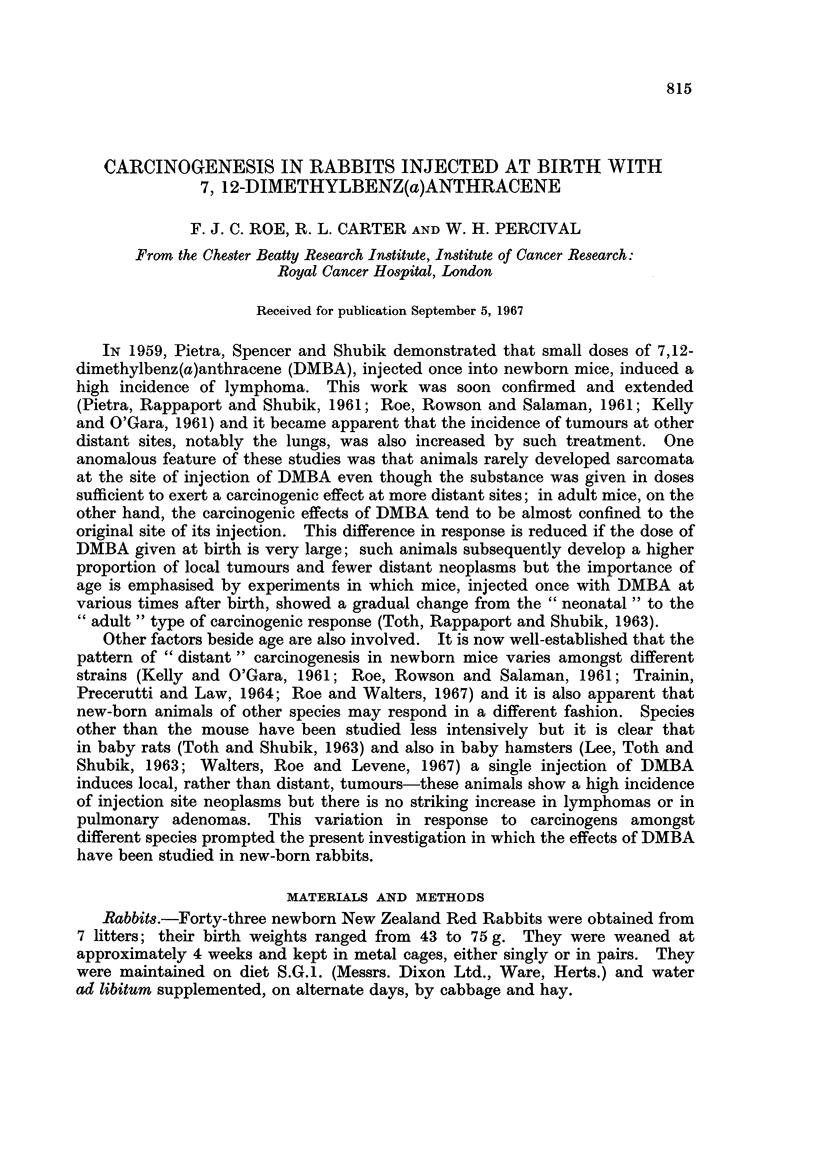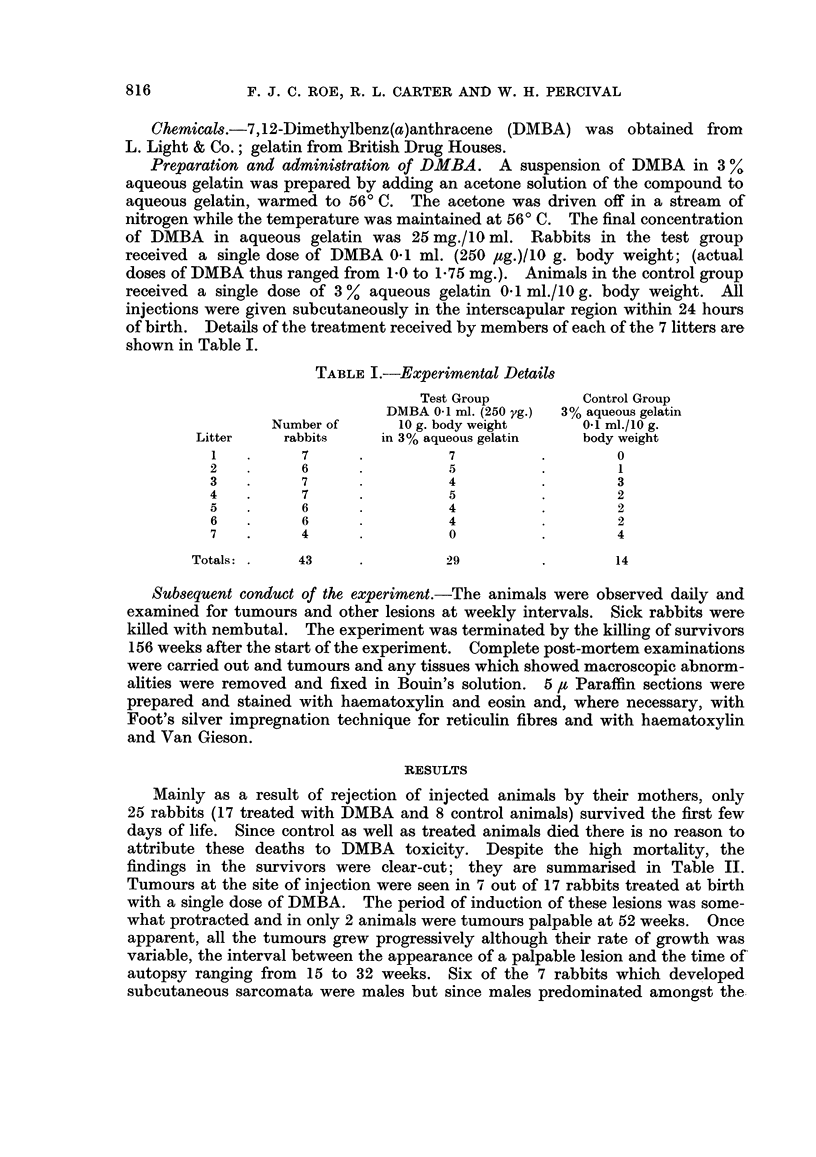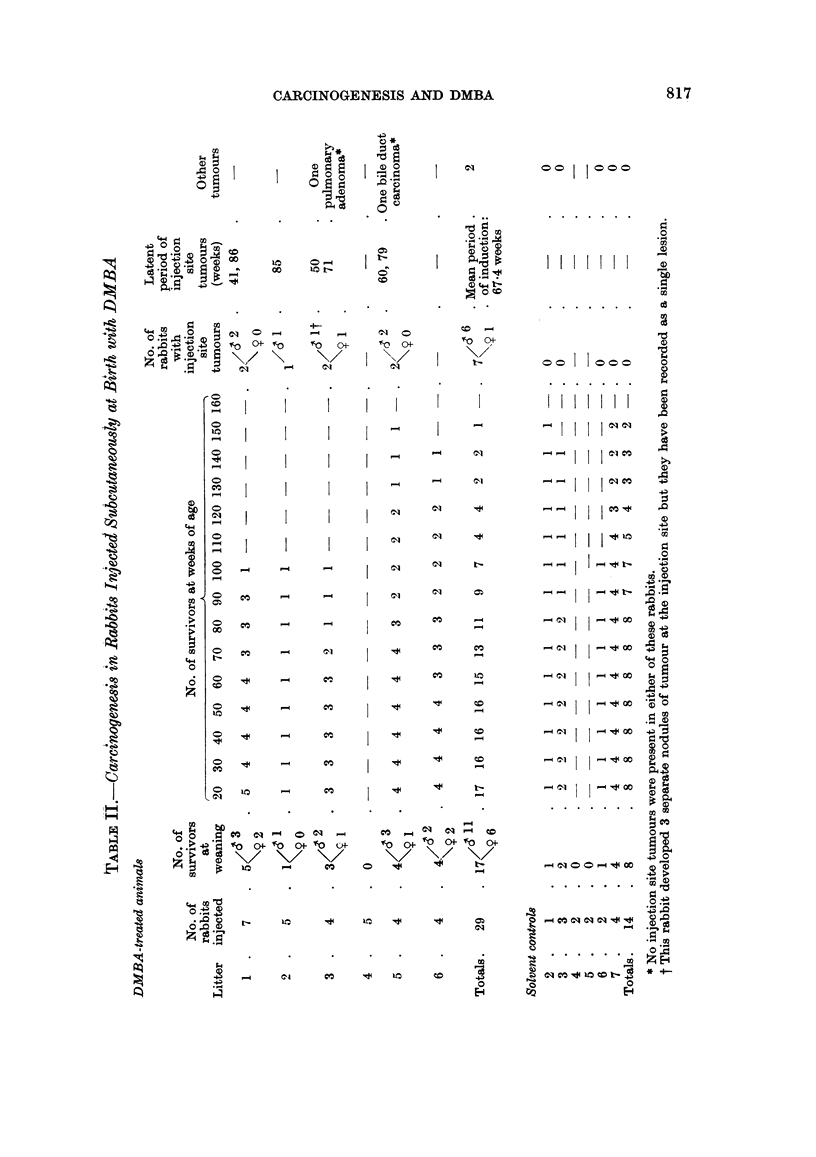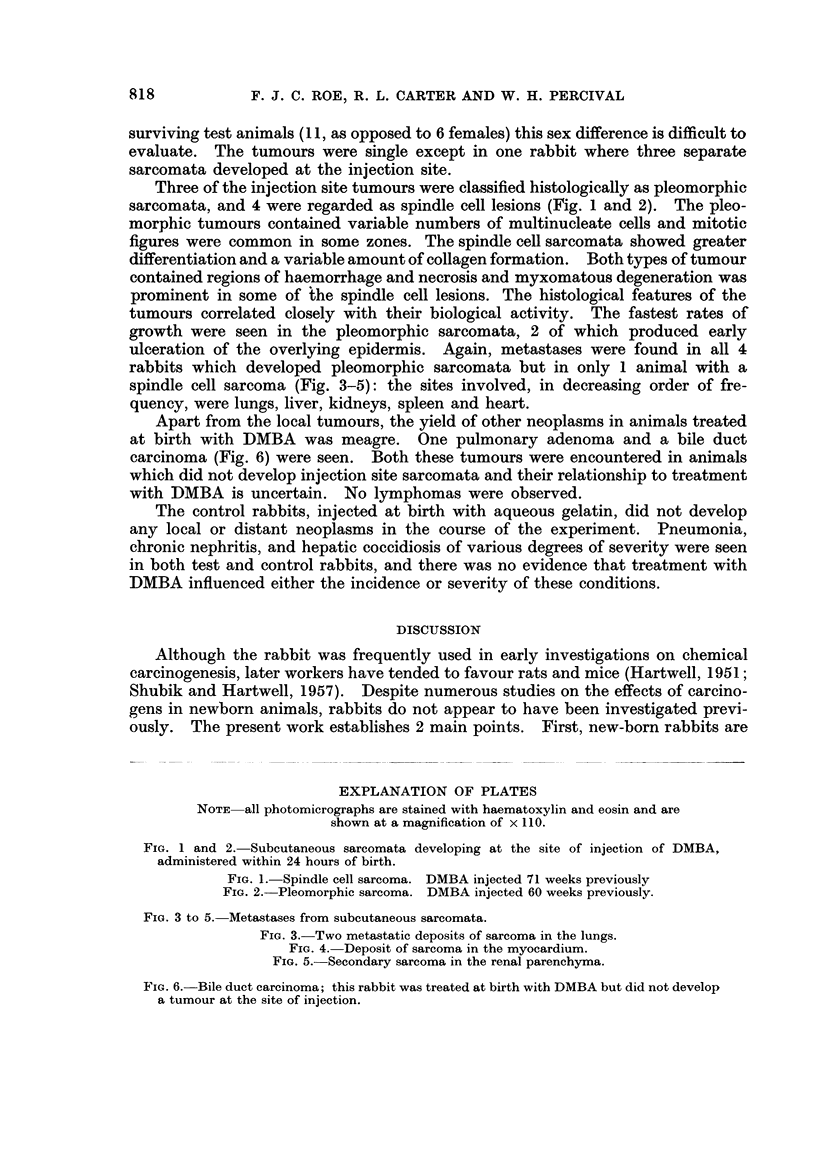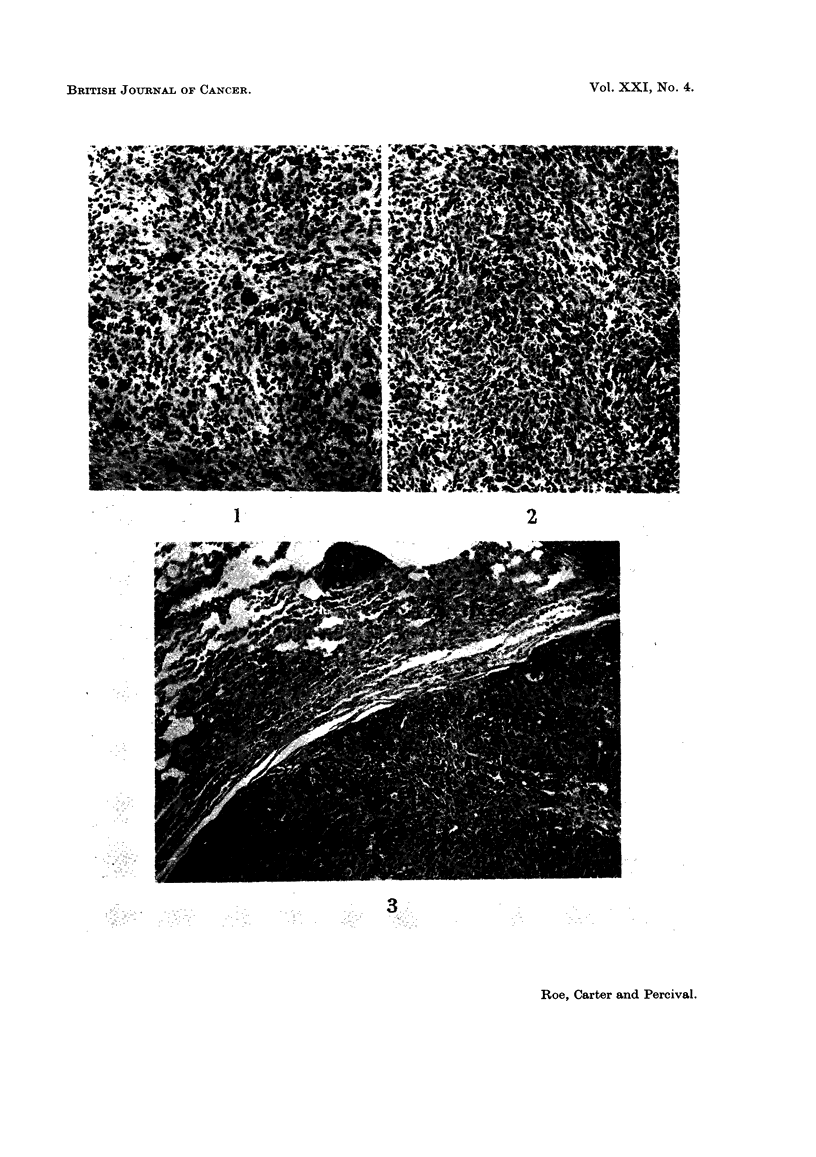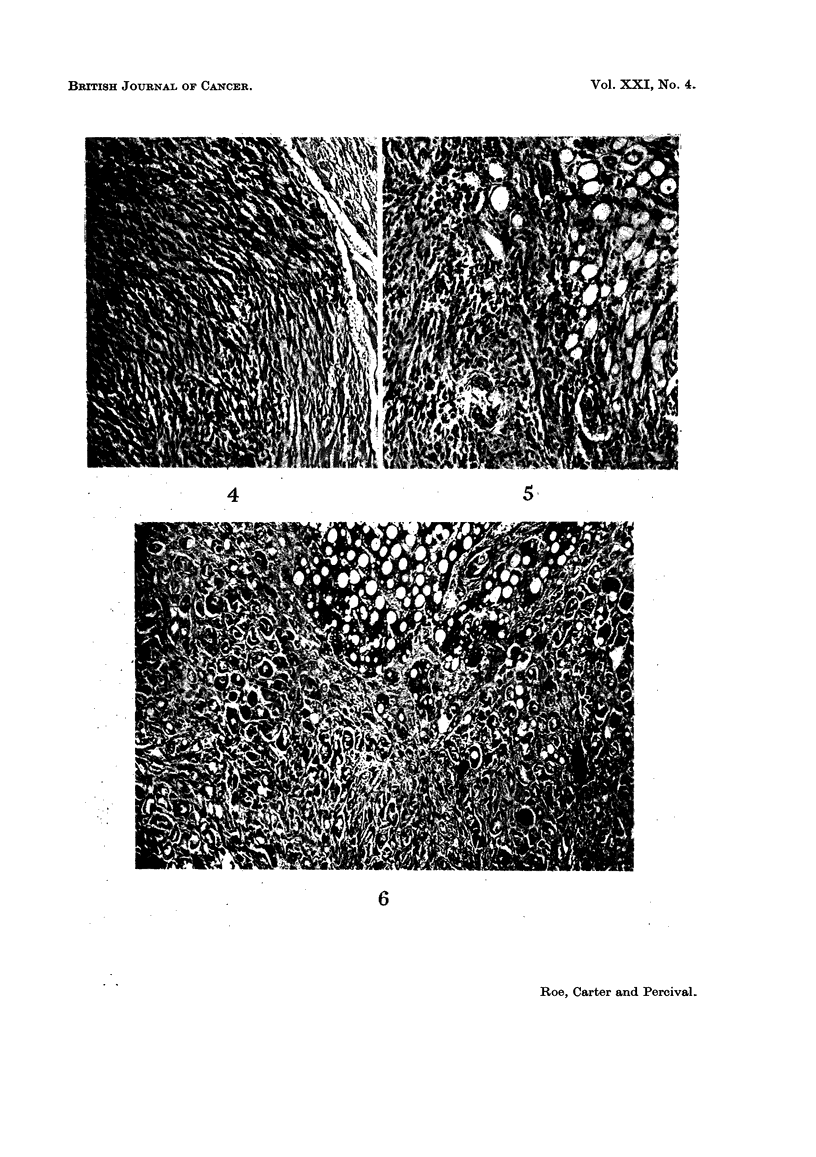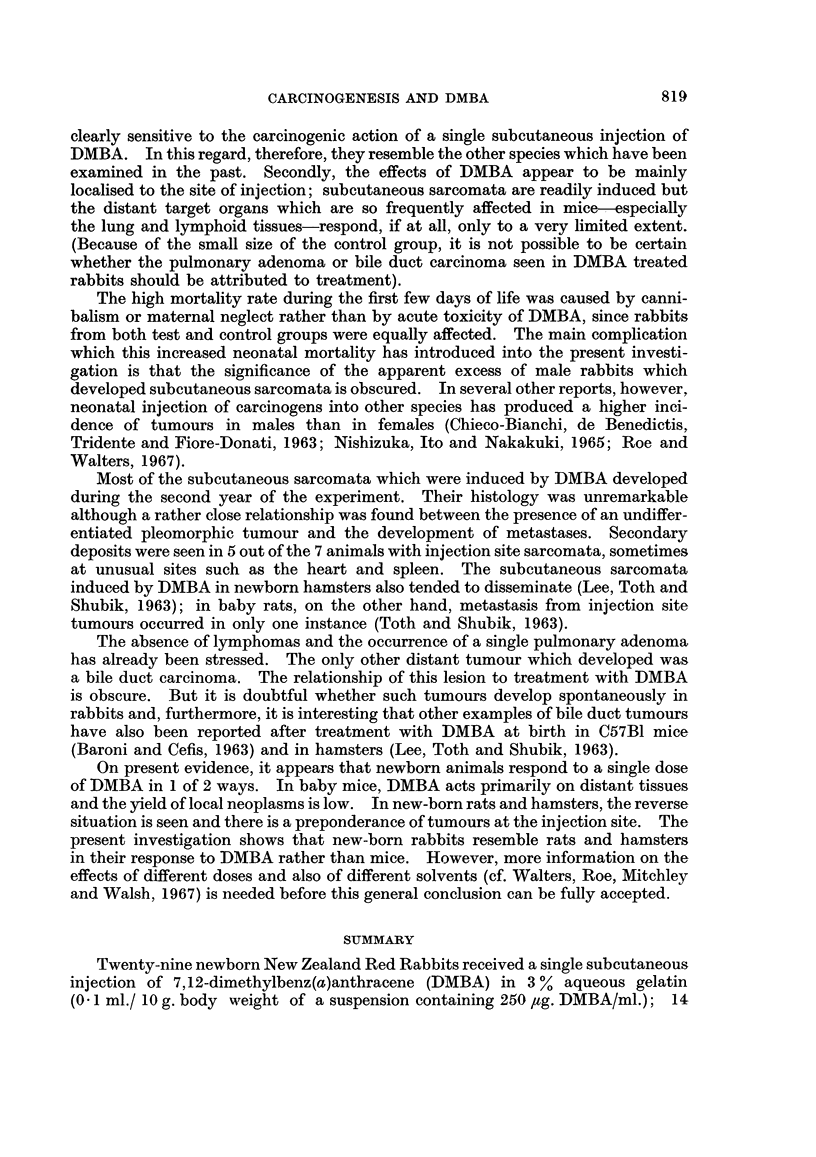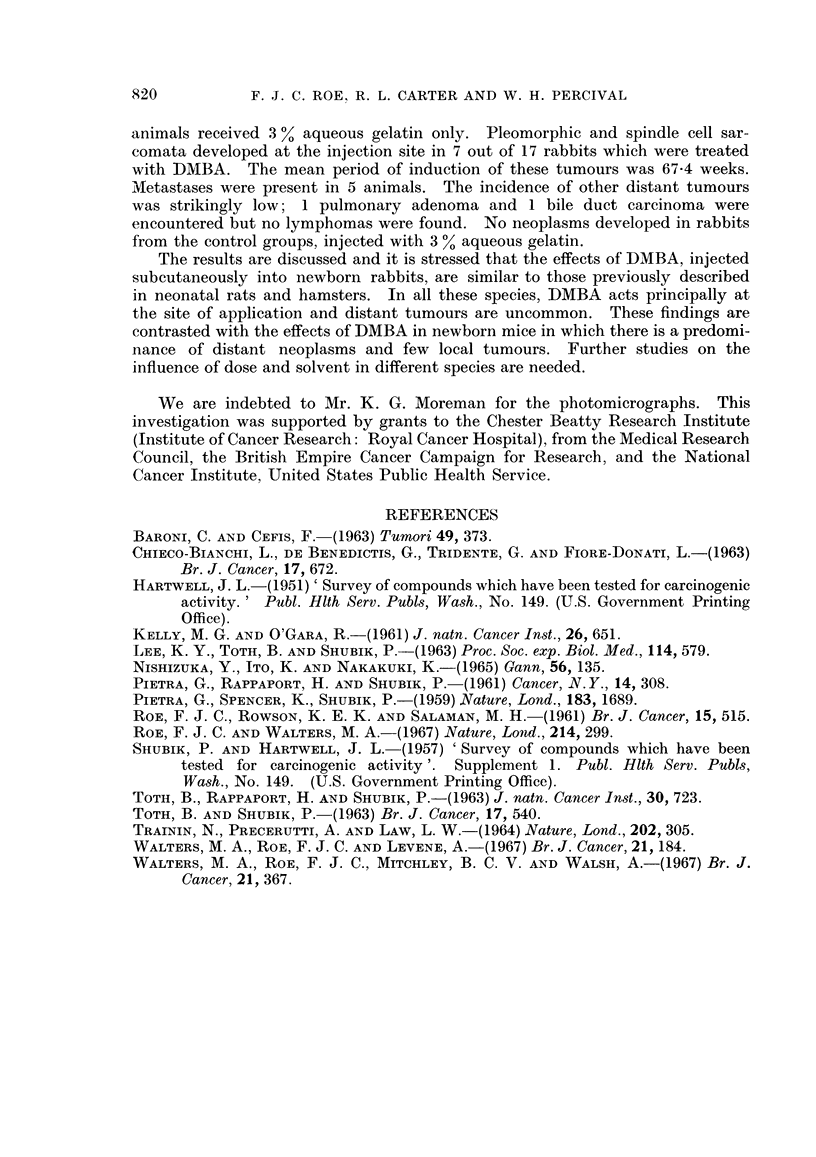# Carcinogenesis in rabbits injected at birth with 7,12-dimethylbenz(a)anthracene.

**DOI:** 10.1038/bjc.1967.96

**Published:** 1967-12

**Authors:** F. J. Roe, R. L. Carter, W. H. Percival

## Abstract

**Images:**


### CARCINOGENESIS IN RABBITS INJECTED AT BIRTH WITH 7, 12-DIMETHYLBENZ(*a*)ANTHRACENE

F. J. C. ROE, R. L. CARTER AND W. H. PERCIVAL

*From the Chester Beatty Research Institute, Institute of Cancer Research: Royal Cancer Hospital, London*

Received for publication September 5, 1967

IN 1959, Pietra, Spencer and Shubik demonstrated that small doses of 7,12-dimethylbenz(*a*)anthracene (DMBA), injected once into newborn mice, induced a high incidence of lymphoma. This work was soon confirmed and extended (Pietra, Rappaport and Shubik, 1961; Roe, Rowson and Salaman, 1961; Kelly and O'Gara, 1961) and it became apparent that the incidence of tumours at other distant sites, notably the lungs, was also increased by such treatment. One anomalous feature of these studies was that animals rarely developed sarcomata at the site of injection of DMBA even though the substance was given in doses sufficient to exert a carcinogenic effect at more distant sites; in adult mice, on the other hand, the carcinogenic effects of DMBA tend to be almost confined to the original site of its injection. This difference in response is reduced if the dose of DMBA given at birth is very large; such animals subsequently develop a higher proportion of local tumours and fewer distant neoplasms but the importance of age is emphasised by experiments in which mice, injected once with DMBA at various times after birth, showed a gradual change from the " neonatal " to the " adult " type of carcinogenic response (Toth, Rappaport and Shubik, 1963).

Other factors beside age are also involved. It is now well-established that the pattern of " distant " carcinogenesis in newborn mice varies amongst different strains (Kelly and O'Gara, 1961; Roe, Rowson and Salaman, 1961; Trainin, Precerutti and Law, 1964; Roe and Walters, 1967) and it is also apparent that new-born animals of other species may respond in a different fashion. Species other than the mouse have been studied less intensively but it is clear that in baby rats (Toth and Shubik, 1963) and also in baby hamsters (Lee, Toth and Shubik, 1963; Walters, Roe and Levene, 1967) a single injection of DMBA induces local, rather than distant, tumours—these animals show a high incidence of injection site neoplasms but there is no striking increase in lymphomas or in pulmonary adenomas. This variation in response to carcinogens amongst different species prompted the present investigation in which the effects of DMBA have been studied in new-born rabbits.

### MATERIALS AND METHODS

*Rabbits.*—Forty-three newborn New Zealand Red Rabbits were obtained from 7 litters; their birth weights ranged from 43 to 75 g. They were weaned at approximately 4 weeks and kept in metal cages, either singly or in pairs. They were maintained on diet S.G.1. (Messrs. Dixon Ltd., Ware, Herts.) and water *ad libitum* supplemented, on alternate days, by cabbage and hay.

*Chemicals.*—7,12-Dimethylbenz(*a*)anthracene (DMBA) was obtained from L. Light & Co.; gelatin from British Drug Houses.

*Preparation and administration of DMBA.* A suspension of DMBA in 3% aqueous gelatin was prepared by adding an acetone solution of the compound to aqueous gelatin, warmed to 56° C. The acetone was driven off in a stream of nitrogen while the temperature was maintained at 56° C. The final concentration of DMBA in aqueous gelatin was 25 mg./10 ml. Rabbits in the test group received a single dose of DMBA 0·1 ml. (250 $\mu$g.)/10 g. body weight; (actual doses of DMBA thus ranged from 1·0 to 1·75 mg.). Animals in the control group received a single dose of 3% aqueous gelatin 0·1 ml./10 g. body weight. All injections were given subcutaneously in the interscapular region within 24 hours of birth. Details of the treatment received by members of each of the 7 litters are shown in Table I.

TABLE I.—*Experimental Details*

| Litter | Number of rabbits | Test Group DMBA 0·1 ml. (250 $\gamma$g.) 10 g. body weight in 3% aqueous gelatin | Control Group 3% aqueous gelatin 0·1 ml./10 g. body weight |
|---|---|---|---|
| 1 | 7 | 7 | 0 |
| 2 | 6 | 5 | 1 |
| 3 | 7 | 4 | 3 |
| 4 | 7 | 5 | 2 |
| 5 | 6 | 4 | 2 |
| 6 | 6 | 4 | 2 |
| 7 | 4 | 0 | 4 |
| Totals: | 43 | 29 | 14 |

*Subsequent conduct of the experiment.*—The animals were observed daily and examined for tumours and other lesions at weekly intervals. Sick rabbits were killed with nembutal. The experiment was terminated by the killing of survivors 156 weeks after the start of the experiment. Complete post-mortem examinations were carried out and tumours and any tissues which showed macroscopic abnormalities were removed and fixed in Bouin's solution. 5 $\mu$ Paraffin sections were prepared and stained with haematoxylin and eosin and, where necessary, with Foot's silver impregnation technique for reticulin fibres and with haematoxylin and Van Gieson.

### RESULTS

Mainly as a result of rejection of injected animals by their mothers, only 25 rabbits (17 treated with DMBA and 8 control animals) survived the first few days of life. Since control as well as treated animals died there is no reason to attribute these deaths to DMBA toxicity. Despite the high mortality, the findings in the survivors were clear-cut; they are summarised in Table II. Tumours at the site of injection were seen in 7 out of 17 rabbits treated at birth with a single dose of DMBA. The period of induction of these lesions was somewhat protracted and in only 2 animals were tumours palpable at 52 weeks. Once apparent, all the tumours grew progressively although their rate of growth was variable, the interval between the appearance of a palpable lesion and the time of autopsy ranging from 15 to 32 weeks. Six of the 7 rabbits which developed subcutaneous sarcomata were males but since males predominated amongst the

TABLE II.—*Carcinogenesis in Rabbits Injected Subcutaneously at Birth with DMBA*

| Litter | No. of rabbits injected | No. of survivors at weaning | No. of survivors at weeks of age: 20 | 30 | 40 | 50 | 60 | 70 | 80 | 90 | 100 | 110 | 120 | 130 | 140 | 150 | 160 | No. of rabbits with injection site tumours | Latent period of injection site tumours (weeks) | Other tumours |
|---|---|---|---|---|---|---|---|---|---|---|---|---|---|---|---|---|---|---|---|---|
| *DMBA-treated animals* | | | | | | | | | | | | | | | | | | | | |
| 1 | 7 | 5 (♂ 3, ♀ 2) | 5 | 4 | 4 | 4 | 4 | 3 | 3 | 3 | 1 | — | — | — | — | — | — | 2 (♂ 2, ♀ 0) | 41, 86 | — |
| 2 | 5 | 1 (♂ 1, ♀ 0) | 1 | 1 | 1 | 1 | 1 | 1 | 1 | 1 | 1 | — | — | — | — | — | — | 1 (♂ 1) | 85 | — |
| 3 | 4 | 3 (♂ 2, ♀ 1) | 3 | 3 | 3 | 3 | 3 | 2 | 1 | 1 | 1 | — | — | — | — | — | — | 2 (♂ 1†, ♀ 1) | 50<br>71 | One pulmonary adenoma* |
| 4 | 5 | 0 | — | — | — | — | — | — | — | — | — | — | — | — | — | — | — | — | — | — |
| 5 | 4 | 4 (♂ 3, ♀ 1) | 4 | 4 | 4 | 4 | 4 | 4 | 3 | 2 | 2 | 2 | 2 | 1 | 1 | 1 | — | 2 (♂ 2, ♀ 0) | 60, 79 | One bile duct carcinoma* |
| 6 | 4 | 4 (♂ 2, ♀ 2) | 4 | 4 | 4 | 4 | 3 | 3 | 3 | 2 | 2 | 2 | 2 | 1 | 1 | — | — | — | — | — |
| Totals | 29 | 17 (♂ 11, ♀ 6) | 17 | 16 | 16 | 16 | 15 | 13 | 11 | 9 | 7 | 4 | 4 | 2 | 2 | 1 | — | 7 (♂ 6, ♀ 1) | Mean period of induction: 67·4 weeks | 2 |
| *Solvent controls* | | | | | | | | | | | | | | | | | | | | |
| 2 | 1 | 1 | 1 | 1 | 1 | 1 | 1 | 1 | 1 | 1 | 1 | 1 | 1 | 1 | 1 | 1 | — | 0 | — | 0 |
| 3 | 3 | 2 | 2 | 2 | 2 | 2 | 2 | 2 | 2 | 1 | 1 | 1 | 1 | 1 | 1 | — | — | 0 | — | 0 |
| 4 | 2 | 0 | — | — | — | — | — | — | — | — | — | — | — | — | — | — | — | — | — | — |
| 5 | 2 | 0 | — | — | — | — | — | — | — | — | — | — | — | — | — | — | — | — | — | — |
| 6 | 2 | 1 | 1 | 1 | 1 | 1 | 1 | 1 | 1 | 1 | 1 | — | — | — | — | — | — | 0 | — | 0 |
| 7 | 4 | 4 | 4 | 4 | 4 | 4 | 4 | 4 | 4 | 4 | 4 | 4 | 3 | 2 | 2 | 2 | — | 0 | — | 0 |
| Totals | 14 | 8 | 8 | 8 | 8 | 8 | 8 | 8 | 8 | 7 | 7 | 5 | 4 | 3 | 3 | 2 | — | 0 | — | 0 |

* No injection site tumours were present in either of these rabbits.

† This rabbit developed 3 separate nodules of tumour at the injection site but they have been recorded as a single lesion.

surviving test animals (11, as opposed to 6 females) this sex difference is difficult to evaluate. The tumours were single except in one rabbit where three separate sarcomata developed at the injection site.

Three of the injection site tumours were classified histologically as pleomorphic sarcomata, and 4 were regarded as spindle cell lesions (Fig. 1 and 2). The pleomorphic tumours contained variable numbers of multinucleate cells and mitotic figures were common in some zones. The spindle cell sarcomata showed greater differentiation and a variable amount of collagen formation. Both types of tumour contained regions of haemorrhage and necrosis and myxomatous degeneration was prominent in some of the spindle cell lesions. The histological features of the tumours correlated closely with their biological activity. The fastest rates of growth were seen in the pleomorphic sarcomata, 2 of which produced early ulceration of the overlying epidermis. Again, metastases were found in all 4 rabbits which developed pleomorphic sarcomata but in only 1 animal with a spindle cell sarcoma (Fig. 3–5): the sites involved, in decreasing order of frequency, were lungs, liver, kidneys, spleen and heart.

Apart from the local tumours, the yield of other neoplasms in animals treated at birth with DMBA was meagre. One pulmonary adenoma and a bile duct carcinoma (Fig. 6) were seen. Both these tumours were encountered in animals which did not develop injection site sarcomata and their relationship to treatment with DMBA is uncertain. No lymphomas were observed.

The control rabbits, injected at birth with aqueous gelatin, did not develop any local or distant neoplasms in the course of the experiment. Pneumonia, chronic nephritis, and hepatic coccidiosis of various degrees of severity were seen in both test and control rabbits, and there was no evidence that treatment with DMBA influenced either the incidence or severity of these conditions.

### DISCUSSION

Although the rabbit was frequently used in early investigations on chemical carcinogenesis, later workers have tended to favour rats and mice (Hartwell, 1951; Shubik and Hartwell, 1957). Despite numerous studies on the effects of carcinogens in newborn animals, rabbits do not appear to have been investigated previously. The present work establishes 2 main points. First, new-born rabbits are

---

### EXPLANATION OF PLATES

NOTE—all photomicrographs are stained with haematoxylin and eosin and are shown at a magnification of × 110.

FIG. 1 and 2.—Subcutaneous sarcomata developing at the site of injection of DMBA, administered within 24 hours of birth.

FIG. 1.—Spindle cell sarcoma. DMBA injected 71 weeks previously

FIG. 2.—Pleomorphic sarcoma. DMBA injected 60 weeks previously.

FIG. 3 to 5.—Metastases from subcutaneous sarcomata.

FIG. 3.—Two metastatic deposits of sarcoma in the lungs.

FIG. 4.—Deposit of sarcoma in the myocardium.

FIG. 5.—Secondary sarcoma in the renal parenchyma.

FIG. 6.—Bile duct carcinoma; this rabbit was treated at birth with DMBA but did not develop a tumour at the site of injection.

1

2

3

4

5

6

clearly sensitive to the carcinogenic action of a single subcutaneous injection of DMBA. In this regard, therefore, they resemble the other species which have been examined in the past. Secondly, the effects of DMBA appear to be mainly localised to the site of injection; subcutaneous sarcomata are readily induced but the distant target organs which are so frequently affected in mice—especially the lung and lymphoid tissues—respond, if at all, only to a very limited extent. (Because of the small size of the control group, it is not possible to be certain whether the pulmonary adenoma or bile duct carcinoma seen in DMBA treated rabbits should be attributed to treatment).

The high mortality rate during the first few days of life was caused by cannibalism or maternal neglect rather than by acute toxicity of DMBA, since rabbits from both test and control groups were equally affected. The main complication which this increased neonatal mortality has introduced into the present investigation is that the significance of the apparent excess of male rabbits which developed subcutaneous sarcomata is obscured. In several other reports, however, neonatal injection of carcinogens into other species has produced a higher incidence of tumours in males than in females (Chieco-Bianchi, de Benedictis, Tridente and Fiore-Donati, 1963; Nishizuka, Ito and Nakakuki, 1965; Roe and Walters, 1967).

Most of the subcutaneous sarcomata which were induced by DMBA developed during the second year of the experiment. Their histology was unremarkable although a rather close relationship was found between the presence of an undifferentiated pleomorphic tumour and the development of metastases. Secondary deposits were seen in 5 out of the 7 animals with injection site sarcomata, sometimes at unusual sites such as the heart and spleen. The subcutaneous sarcomata induced by DMBA in newborn hamsters also tended to disseminate (Lee, Toth and Shubik, 1963); in baby rats, on the other hand, metastasis from injection site tumours occurred in only one instance (Toth and Shubik, 1963).

The absence of lymphomas and the occurrence of a single pulmonary adenoma has already been stressed. The only other distant tumour which developed was a bile duct carcinoma. The relationship of this lesion to treatment with DMBA is obscure. But it is doubtful whether such tumours develop spontaneously in rabbits and, furthermore, it is interesting that other examples of bile duct tumours have also been reported after treatment with DMBA at birth in C57Bl mice (Baroni and Cefis, 1963) and in hamsters (Lee, Toth and Shubik, 1963).

On present evidence, it appears that newborn animals respond to a single dose of DMBA in 1 of 2 ways. In baby mice, DMBA acts primarily on distant tissues and the yield of local neoplasms is low. In new-born rats and hamsters, the reverse situation is seen and there is a preponderance of tumours at the injection site. The present investigation shows that new-born rabbits resemble rats and hamsters in their response to DMBA rather than mice. However, more information on the effects of different doses and also of different solvents (cf. Walters, Roe, Mitchley and Walsh, 1967) is needed before this general conclusion can be fully accepted.

### SUMMARY

Twenty-nine newborn New Zealand Red Rabbits received a single subcutaneous injection of 7,12-dimethylbenz(*a*)anthracene (DMBA) in 3% aqueous gelatin (0·1 ml./ 10 g. body weight of a suspension containing 250 μg. DMBA/ml.); 14

animals received 3% aqueous gelatin only. Pleomorphic and spindle cell sarcomata developed at the injection site in 7 out of 17 rabbits which were treated with DMBA. The mean period of induction of these tumours was 67·4 weeks. Metastases were present in 5 animals. The incidence of other distant tumours was strikingly low; 1 pulmonary adenoma and 1 bile duct carcinoma were encountered but no lymphomas were found. No neoplasms developed in rabbits from the control groups, injected with 3% aqueous gelatin.

The results are discussed and it is stressed that the effects of DMBA, injected subcutaneously into newborn rabbits, are similar to those previously described in neonatal rats and hamsters. In all these species, DMBA acts principally at the site of application and distant tumours are uncommon. These findings are contrasted with the effects of DMBA in newborn mice in which there is a predominance of distant neoplasms and few local tumours. Further studies on the influence of dose and solvent in different species are needed.

We are indebted to Mr. K. G. Moreman for the photomicrographs. This investigation was supported by grants to the Chester Beatty Research Institute (Institute of Cancer Research: Royal Cancer Hospital), from the Medical Research Council, the British Empire Cancer Campaign for Research, and the National Cancer Institute, United States Public Health Service.

### REFERENCES

Baroni, C. and Cefis, F.—(1963) *Tumori* **49,** 373.

Chieco-Bianchi, L., de Benedictis, G., Tridente, G. and Fiore-Donati, L.—(1963) *Br. J. Cancer,* **17,** 672.

Hartwell, J. L.—(1951) 'Survey of compounds which have been tested for carcinogenic activity.' *Publ. Hlth Serv. Publs, Wash.,* No. 149. (U.S. Government Printing Office).

Kelly, M. G. and O'Gara, R.—(1961) *J. natn. Cancer Inst.,* **26,** 651.

Lee, K. Y., Toth, B. and Shubik, P.—(1963) *Proc. Soc. exp. Biol. Med.,* **114,** 579.

Nishizuka, Y., Ito, K. and Nakakuki, K.—(1965) *Gann,* **56,** 135.

Pietra, G., Rappaport, H. and Shubik, P.—(1961) *Cancer, N.Y.,* **14,** 308.

Pietra, G., Spencer, K., Shubik, P.—(1959) *Nature, Lond.,* **183,** 1689.

Roe, F. J. C., Rowson, K. E. K. and Salaman, M. H.—(1961) *Br. J. Cancer,* **15,** 515.

Roe, F. J. C. and Walters, M. A.—(1967) *Nature, Lond.,* **214,** 299.

Shubik, P. and Hartwell, J. L.—(1957) 'Survey of compounds which have been tested for carcinogenic activity'. Supplement 1. *Publ. Hlth Serv. Publs, Wash.,* No. 149. (U.S. Government Printing Office).

Toth, B., Rappaport, H. and Shubik, P.—(1963) *J. natn. Cancer Inst.,* **30,** 723.

Toth, B. and Shubik, P.—(1963) *Br. J. Cancer,* **17,** 540.

Trainin, N., Precerutti, A. and Law, L. W.—(1964) *Nature, Lond.,* **202,** 305.

Walters, M. A., Roe, F. J. C. and Levene, A.—(1967) *Br. J. Cancer,* **21,** 184.

Walters, M. A., Roe, F. J. C., Mitchley, B. C. V. and Walsh, A.—(1967) *Br. J. Cancer,* **21,** 367.